# Supplementary material for: The Health Effects of Chocolate and Cocoa: A Systematic Review
Source: Nutrients. 2021 Aug 24;13(9):2909. doi: 10.3390/nu13092909 (PMC8470865; doi:10.3390/nu13092909)
Supplement: Supplementary file 1 [file nutrients-13-02909-s001.zip › nutrients-1303200-supplementary.pdf]

nutrients MDPI

Table S1: Search Strategy

MEDLINE (PubMed) search strategy is shown as follows:	
Search Date: ________	
Search number	Details
#1	Add Search chocolate*[Title/Abstract]
#2	Add Search chocolates[MeSH Terms]
#3	Add Search cocoa[Title/Abstract]
#4	Add Search cocoa[MeSH Terms]
#5	Add Search cacao[Title/Abstract]
#6	Add Search cacaos[MeSH Terms]
#7	Add Search (#1 OR #2 OR #3 OR #4 OR #5 OR #6)
#8	Add Search "clinical study"[Publication Type]
#9	Add Search (#7 AND #8)

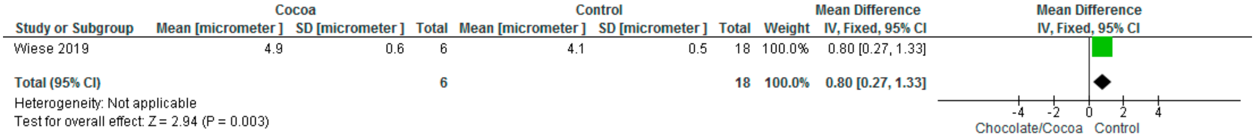

Figure S1. Forest plot of comparison: Chocolate versus control did not show any significant difference in mean of sebum droplet size (micrometer).

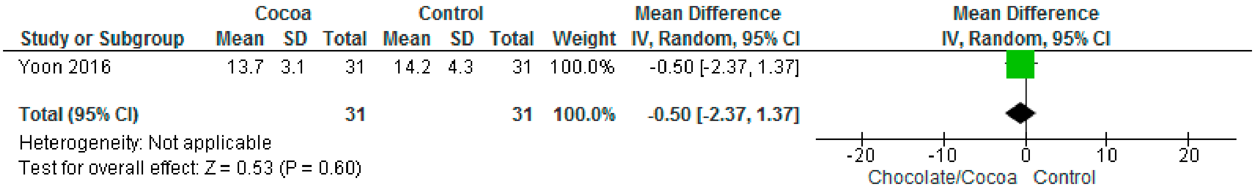

Figure S2. Forest plot of comparison: Chocolate versus control did not show any significant difference in mean of sebum droplet size (micrometer).

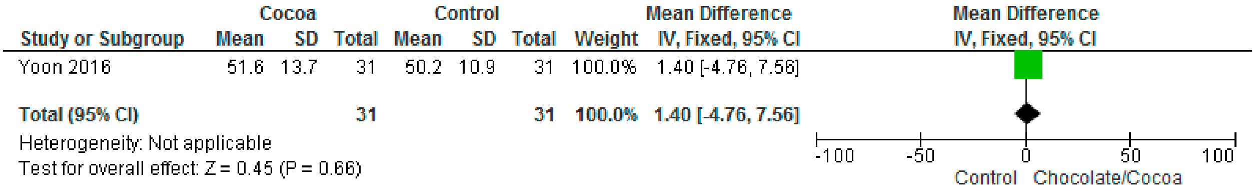

Figure S3. Forest plot of comparison: Chocolate versus control did not show any significant difference in mean of sebum droplet size (micrometer).

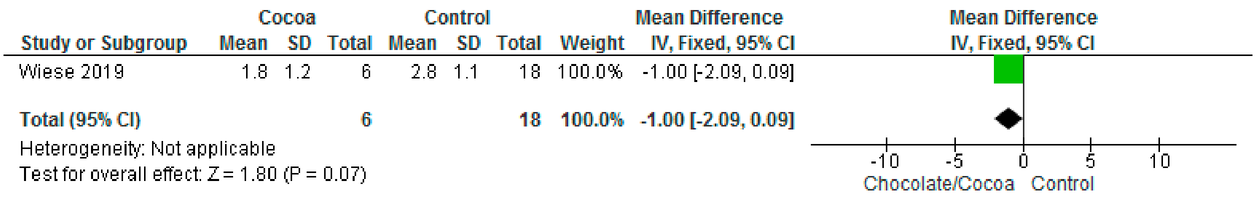

Figure S4. Forest plot of comparison: Chocolate versus control did not show any significant difference in mean of corneocyte damage (verage number of cross-linked damaged corneocyte clusters in stratum cornea under microscope x 1000).

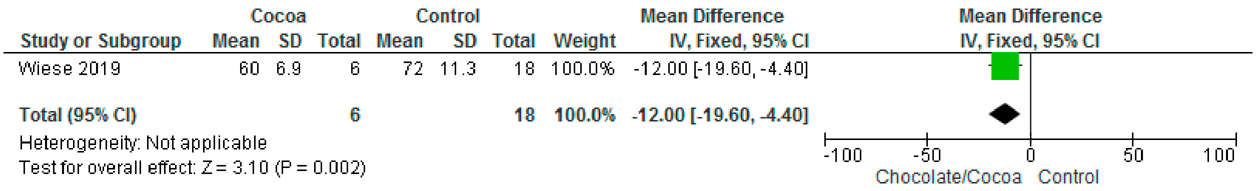

Figure S5. Forest plot of comparison: Chocolate versus control did not show any significant difference in mean of corneocyte exfoliation rate.

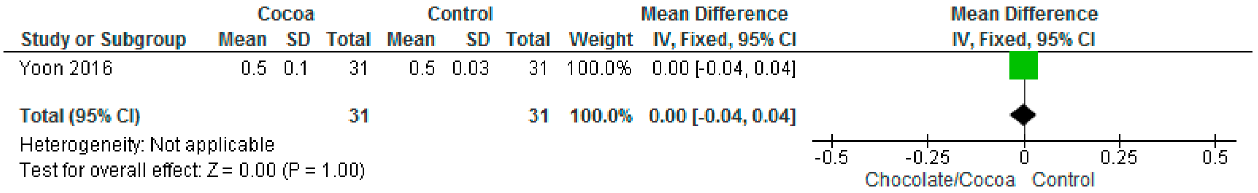

Figure S6. Forest plot of comparison: Chocolate versus control did not show any significant difference in mean of wrinkle severity (Skin roughness measured using transparency profilometry).

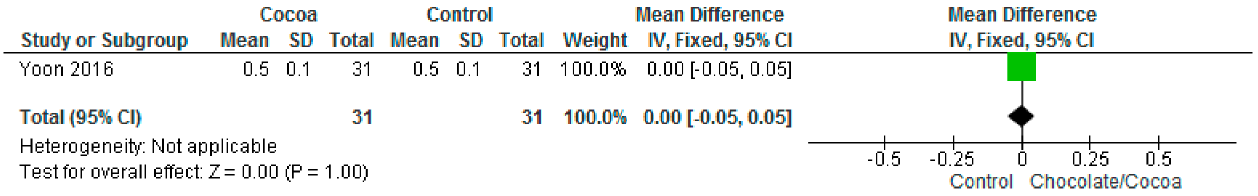

Figure S7. Forest plot of comparison: Chocolate versus control did not show any significant difference in mean of skin elasticity.

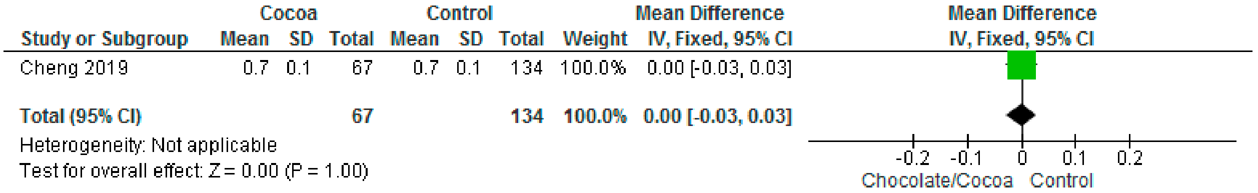

Figure S8. Forest plot of comparison: Chocolate versus control did not show any significant difference in mean of waist hip ratio.

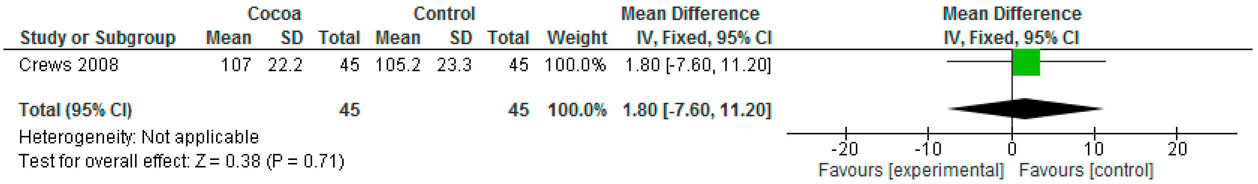

Figure S9. Forest plot of comparison: Chocolate versus control did not show any significant difference in mean of cognitive function: Selective Reminding Test (Immediate free recall)

	Cocoa			Control				Mean Difference
**Study or Subgroup**	**Mean**	**SD**	**Total**	**Mean**	**SD**	**Total**	**Weight**	**IV, Fixed, 95% CI**
Crews 2008	99.1	30.1	45	97.8	31.4	45	100.0%	1.30 [-11.41, 14.01]
**Total (95% CI)**			**45**			**45**	**100.0%**	**1.30 [-11.41, 14.01]**
Heterogeneity: Not applicable								
Test for overall effect: Z = 0.20 (P = 0.84)								

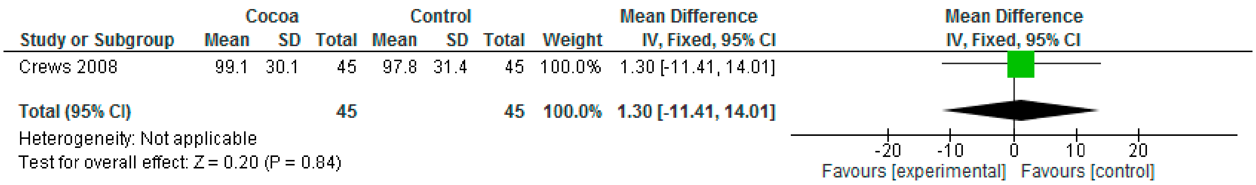

Figure S10. Forest plot of comparison: Chocolate versus control did not show any significant difference in mean of cognitive function: Selective Reminding Test (Long-term storage)

	Cocoa			Control				Mean Difference
**Study or Subgroup**	**Mean**	**SD**	**Total**	**Mean**	**SD**	**Total**	**Weight**	**IV, Fixed, 95% CI**
Crews 2008	15.9	10.3	45	16.6	11.8	45	100.0%	-0.70 [-5.28, 3.88]
**Total (95% CI)**			**45**			**45**	**100.0%**	**-0.70 [-5.28, 3.88]**
Heterogeneity: Not applicable								
Test for overall effect: Z = 0.30 (P = 0.76)								

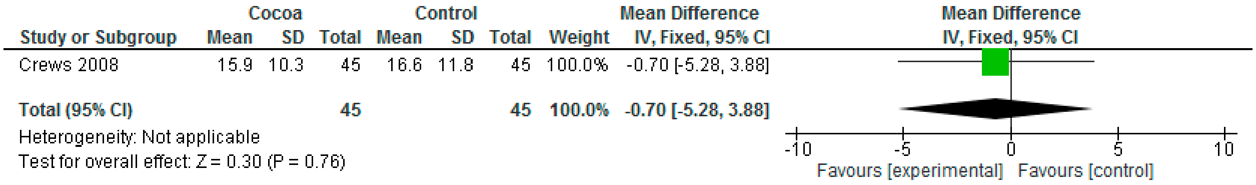

Figure S11. Forest plot of comparison: Chocolate versus control did not show any significant difference in mean of cognitive function: Selective Reminding Test (Short-term recall)

	Cocoa			Control				Mean Difference
**Study or Subgroup**	**Mean**	**SD**	**Total**	**Mean**	**SD**	**Total**	**Weight**	**IV, Fixed, 95% CI**
Crews 2008	91.1	31.5	45	88.6	34.4	45	100.0%	2.50 [-11.13, 16.13]
**Total (95% CI)**			**45**			**45**	**100.0%**	**2.50 [-11.13, 16.13]**
Heterogeneity: Not applicable								
Test for overall effect: Z = 0.36 (P = 0.72)								

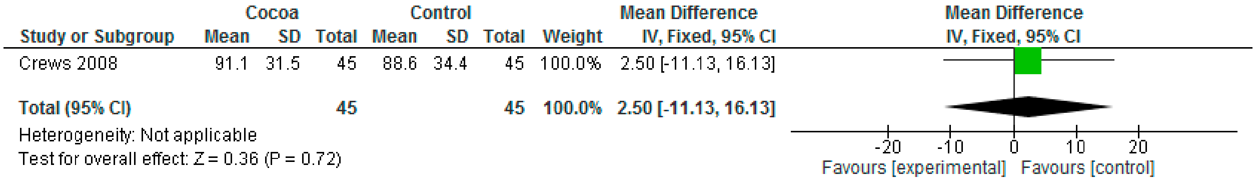

Figure S12. Forest plot of comparison: Chocolate versus control did not show any significant difference in mean of cognitive function: Selective Reminding Test (Long-term retrieval)

	Cocoa			Control				Mean Difference
**Study or Subgroup**	**Mean**	**SD**	**Total**	**Mean**	**SD**	**Total**	**Weight**	**IV, Fixed, 95% CI**
Crews 2008	70.8	36.2	45	64.3	41.4	45	100.0%	6.50 [-9.57, 22.57]
**Total (95% CI)**			**45**			**45**	**100.0%**	**6.50 [-9.57, 22.57]**
Heterogeneity: Not applicable								
Test for overall effect: Z = 0.79 (P = 0.43)								

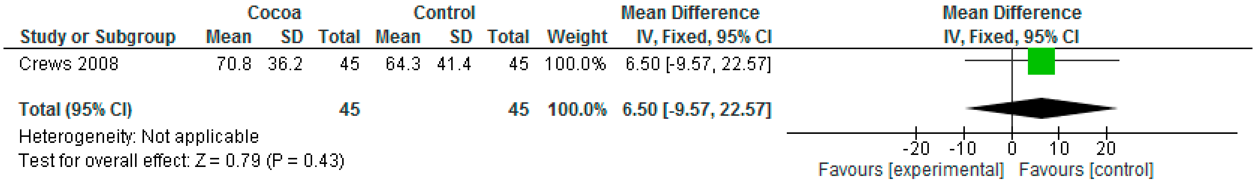

Figure S13. Forest plot of comparison: Chocolate versus control did not show any significant difference in mean of cognitive function: Selective Reminding Test (Consistent long-term retrieval)

Study or Subgroup	Cocoa Mean	Cocoa SD	Cocoa Total	Control Mean	Control SD	Control Total	Weight	Mean Difference IV, Fixed, 95% CI
Crews 2008	20.3	13.1	45	24.2	15.2	45	100.0%	-3.90 [-9.76, 1.96]
**Total (95% CI)**			**45**			**45**	**100.0%**	**-3.90 [-9.76, 1.96]**
Heterogeneity: Not applicable								
Test for overall effect: Z = 1.30 (P = 0.19)								

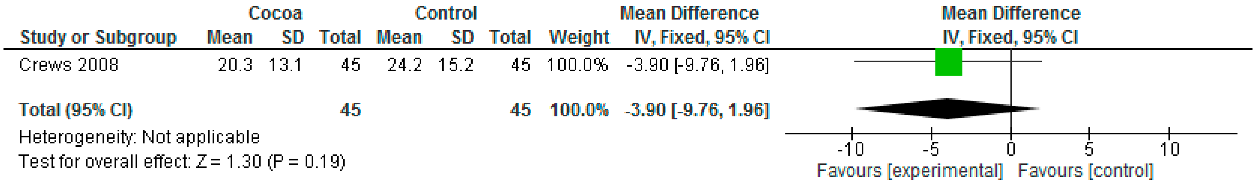

Figure S14. Forest plot of comparison: Chocolate versus control did not show any significant difference in mean of cognitive function: Selective Reminding Test (Random long-term retrieval)

Study or Subgroup	Cocoa Mean	Cocoa SD	Cocoa Total	Control Mean	Control SD	Control Total	Weight	Mean Difference IV, Fixed, 95% CI
Crews 2008	9.4	2.2	45	9.3	2.1	45	100.0%	0.10 [-0.79, 0.99]
**Total (95% CI)**			**45**			**45**	**100.0%**	**0.10 [-0.79, 0.99]**
Heterogeneity: Not applicable								
Test for overall effect: Z = 0.22 (P = 0.83)								

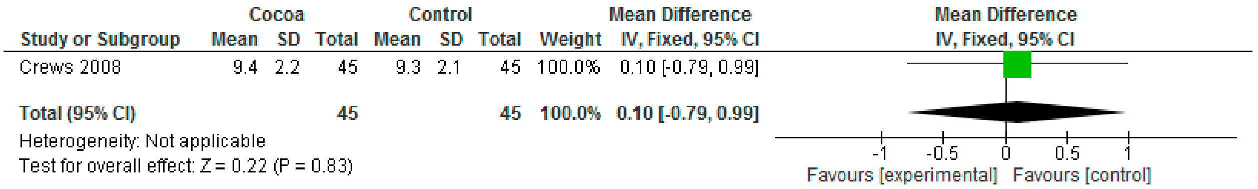

Figure S15. Forest plot of comparison: Chocolate versus control did not show any significant difference in mean of cognitive function: Selective Reminding Test (Cued recall)

Study or Subgroup	Cocoa Mean	Cocoa SD	Cocoa Total	Control Mean	Control SD	Control Total	Weight	Mean Difference IV, Fixed, 95% CI
Crews 2008	8.9	3.4	45	8.9	3.2	45	100.0%	0.00 [-1.36, 1.36]
**Total (95% CI)**			**45**			**45**	**100.0%**	**0.00 [-1.36, 1.36]**
Heterogeneity: Not applicable								
Test for overall effect: Z = 0.00 (P = 1.00)								

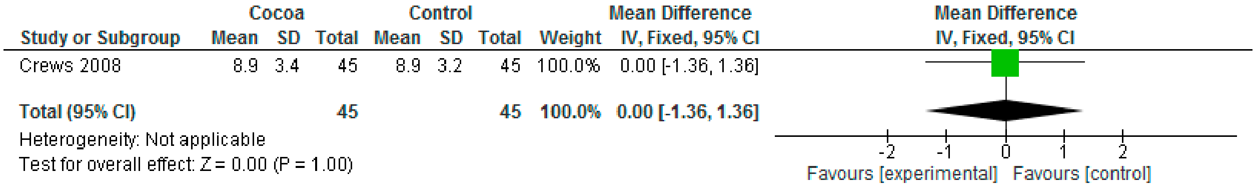

Figure S16. Forest plot of comparison: Chocolate versus control did not show any significant difference in mean of cognitive function: Selective Reminding Test (Delayed free recall)

Study or Subgroup	Cocoa Mean	Cocoa SD	Cocoa Total	Control Mean	Control SD	Control Total	Weight	Mean Difference IV, Fixed, 95% CI
Crews 2008	11.8	0.7	45	11.9	0.4	45	100.0%	-0.10 [-0.34, 0.14]
**Total (95% CI)**			**45**			**45**	**100.0%**	**-0.10 [-0.34, 0.14]**
Heterogeneity: Not applicable								
Test for overall effect: Z = 0.83 (P = 0.41)								

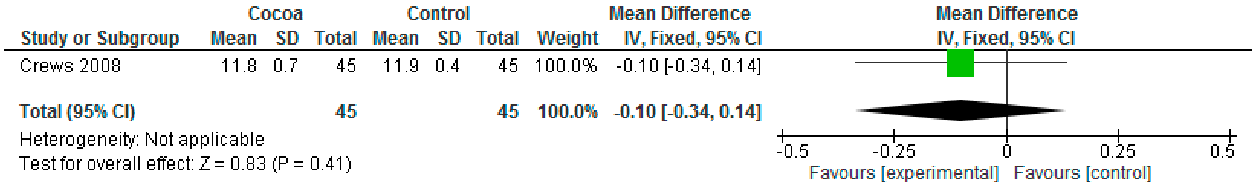

Figure S17. Forest plot of comparison: Chocolate versus control did not show any significant difference in mean of cognitive function: Selective Reminding Test (Delayed recognition)

Study or Subgroup	Cocoa Mean	Cocoa SD	Cocoa Total	Control Mean	Control SD	Control Total	Weight	Mean Difference IV, Fixed, 95% CI
Crews 2008	36.7	5	45	37.8	5.1	45	100.0%	-1.10 [-3.19, 0.99]
**Total (95% CI)**			**45**			**45**	**100.0%**	**-1.10 [-3.19, 0.99]**
Heterogeneity: Not applicable								
Test for overall effect: Z = 1.03 (P = 0.30)								

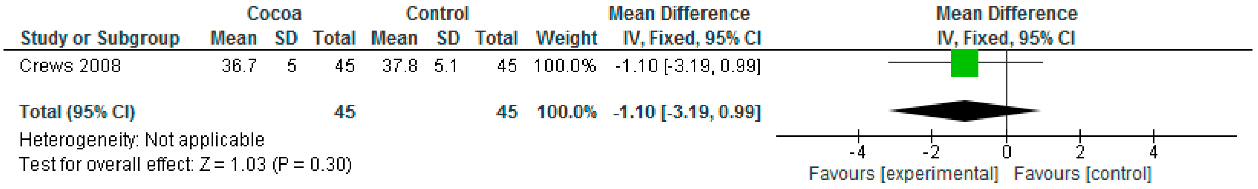

Figure S18. Forest plot of comparison: Chocolate versus control did not show any significant difference in mean of cognitive function: Wechsler Memory Scale-III (Faces I)

Study or Subgroup	Cocoa Mean	Cocoa SD	Cocoa Total	Control Mean	Control SD	Control Total	Weight	Mean Difference IV, Fixed, 95% CI
Crews 2008	38.6	4.1	45	39.2	4	45	100.0%	-0.60 [-2.27, 1.07]
**Total (95% CI)**			**45**			**45**	**100.0%**	**-0.60 [-2.27, 1.07]**

Heterogeneity: Not applicable
Test for overall effect: Z = 0.70 (P = 0.48)

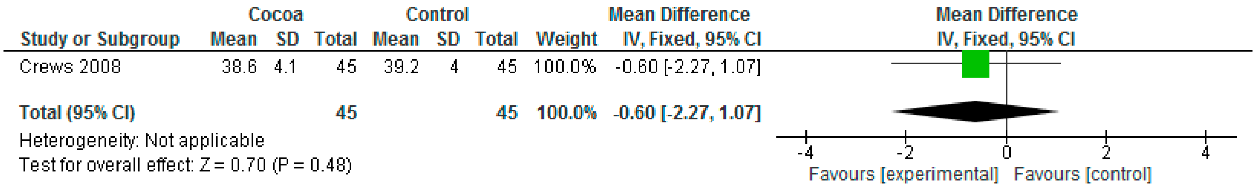

Figure S19. Forest plot of comparison: Chocolate versus control did not show any significant difference in mean of cognitive function: Wechsler Memory Scale-III (Faces II)

Study or Subgroup	Cocoa Mean	Cocoa SD	Cocoa Total	Control Mean	Control SD	Control Total	Weight	Mean Difference IV, Fixed, 95% CI
Crews 2008	68.6	12.9	45	71.9	15.4	45	100.0%	-3.30 [-9.17, 2.57]
**Total (95% CI)**			**45**			**45**	**100.0%**	**-3.30 [-9.17, 2.57]**

Heterogeneity: Not applicable
Test for overall effect: Z = 1.10 (P = 0.27)

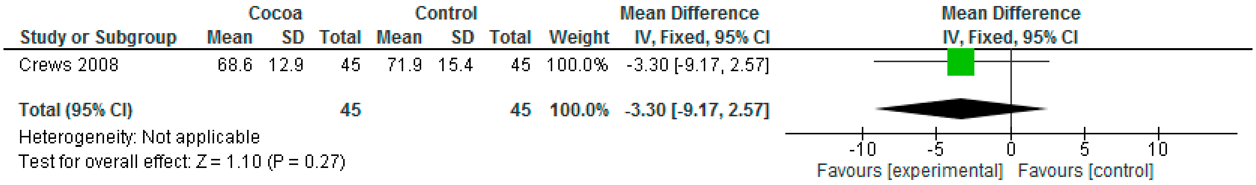

Figure S20. Forest plot of comparison: Chocolate versus control did not show any significant difference in mean of cognitive function: Wechsler Adult Intelligence Scale-III (Digit symbol)

Study or Subgroup	Cocoa Mean	Cocoa SD	Cocoa Total	Control Mean	Control SD	Control Total	Weight	Mean Difference IV, Fixed, 95% CI
Crews 2008	99.3	16.5	45	99.8	16.5	45	100.0%	-0.50 [-7.32, 6.32]
**Total (95% CI)**			**45**			**45**	**100.0%**	**-0.50 [-7.32, 6.32]**

Heterogeneity: Not applicable
Test for overall effect: Z = 0.14 (P = 0.89)

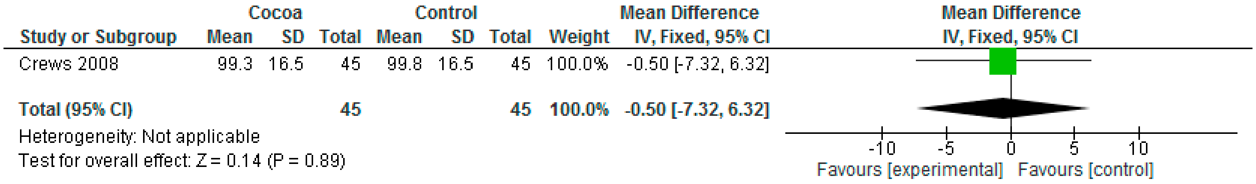

Figure S21. Forest plot of comparison: Chocolate versus control did not show any significant difference in mean of cognitive function: Stroop Color-Word Test (Word)

Study or Subgroup	Cocoa Mean	Cocoa SD	Cocoa Total	Control Mean	Control SD	Control Total	Weight	Mean Difference IV, Fixed, 95% CI
Crews 2008	66.5	12.5	45	68.2	12.7	45	100.0%	-1.70 [-6.91, 3.51]
**Total (95% CI)**			**45**			**45**	**100.0%**	**-1.70 [-6.91, 3.51]**

Heterogeneity: Not applicable
Test for overall effect: Z = 0.64 (P = 0.52)

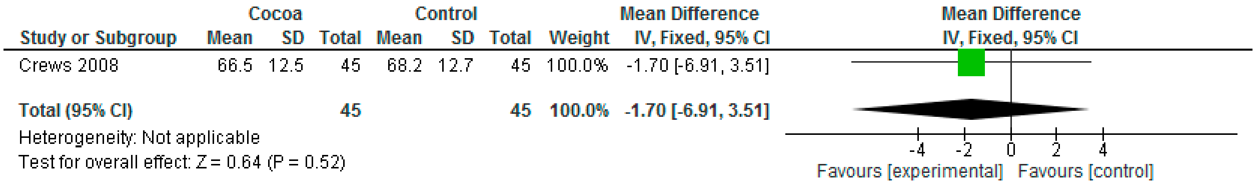

Figure S22. Forest plot of comparison: Chocolate versus control did not show any significant difference in mean of cognitive function: Stroop Color-Word Test (Colour)

Study or Subgroup	Cocoa Mean	Cocoa SD	Cocoa Total	Control Mean	Control SD	Control Total	Weight	Mean Difference IV, Fixed, 95% CI
Crews 2008	35.2	9.2	45	35	10.7	45	100.0%	0.20 [-3.92, 4.32]
**Total (95% CI)**			**45**			**45**	**100.0%**	**0.20 [-3.92, 4.32]**

Heterogeneity: Not applicable
Test for overall effect: Z = 0.10 (P = 0.92)

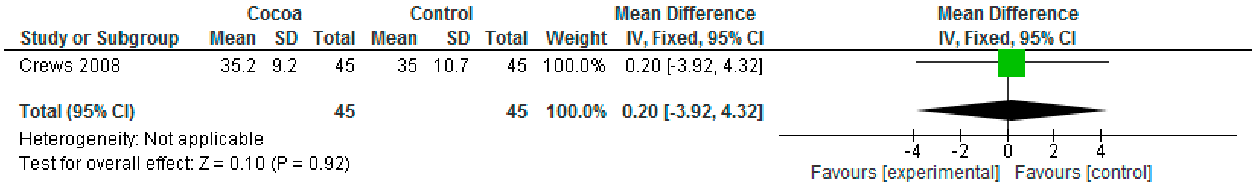

Figure S23. Forest plot of comparison: Chocolate versus control did not show any significant difference in mean of cognitive function: Stroop Color-Word Test (Colour-Word)

Study or Subgroup	Cocoa Mean	Cocoa SD	Cocoa Total	Control Mean	Control SD	Control Total	Weight	Mean Difference IV, Fixed, 95% CI
Crews 2008	17.2	3.7	45	16.4	3.5	43	100.0%	0.80 [-0.70, 2.30]
**Total (95% CI)**			**45**			**43**	**100.0%**	**0.80 [-0.70, 2.30]**
Heterogeneity: Not applicable								
Test for overall effect: Z = 1.04 (P = 0.30)								

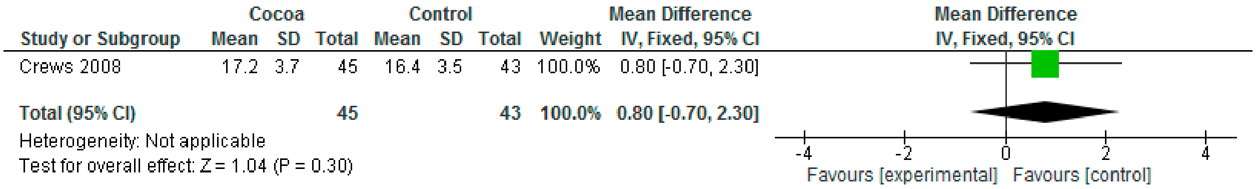

Figure S24. Forest plot of comparison: Chocolate versus control did not show any significant difference in mean of cognitive function: Activation-Deactivation Adjective Check List, General Activation Subscale

Study or Subgroup	Cocoa Mean	Cocoa SD	Cocoa Total	Control Mean	Control SD	Control Total	Weight	Mean Difference IV, Random, 95% CI
Garcia-Yu 2020	3.2	1.06	71	3.12	0.95	66	100.0%	0.08 [-0.26, 0.42]
**Total (95% CI)**			**71**			**66**	**100.0%**	**0.08 [-0.26, 0.42]**
Heterogeneity: Not applicable								
Test for overall effect: Z = 0.47 (P = 0.64)								

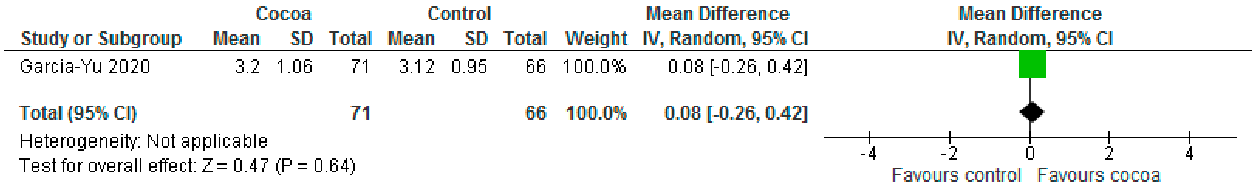

Figure S25. Forest plot of comparison: Chocolate versus control did not show any significant difference in mean of cognitive function: working memory as measured using Digital Span Backward test.

Study or Subgroup	Cocoa Mean	Cocoa SD	Cocoa Total	Control Mean	Control SD	Control Total	Weight	Mean Difference IV, Random, 95% CI
Garcia-Yu 2020	78.1	14	71	77.2	14.9	66	100.0%	0.90 [-3.95, 5.75]
**Total (95% CI)**			**71**			**66**	**100.0%**	**0.90 [-3.95, 5.75]**
Heterogeneity: Not applicable								
Test for overall effect: Z = 0.36 (P = 0.72)								

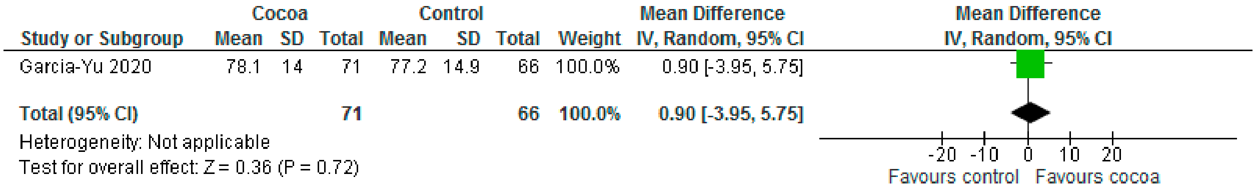

Figure S26. Forest plot of comparison: Chocolate versus control did not show any significant difference in mean of quality of life: EQ-VAS scale (0-100)
